# Obesity prevention practices in early care and education settings: an adaptive implementation trial

**DOI:** 10.1186/s13012-021-01185-1

**Published:** 2022-03-18

**Authors:** Taren Swindle, Julie M. Rutledge, James P. Selig, Jacob Painter, Dong Zhang, Janna Martin, Susan L. Johnson, Leanne Whiteside-Mansell, Daniel Almirall, Tracey Barnett-McElwee, Geoff M. Curran

**Affiliations:** 1 Department of Family and Preventive Medicine, University of Arkansas for Medical Sciences, 4301 W. Markham St, #530, Little Rock, AR 72205-7199 USA; 2 ENRICH Center, College of Applied and Natural Sciences, Louisiana Tech University, Ruston, USA; 3 College of Public Health, Department of Biostatistics, University of Arkansas for Medical Sciences, Little Rock, USA; 4 College of Pharmacy, Division of Pharmaceutical Evaluation & Policy, University of Arkansas for Medical Sciences, Little Rock, USA; 5 Department of Pediatrics, University of Colorado Anschutz Medical Campus, 12700 East 19th Avenue Box C225, Aurora, CO 80045 USA; 6 Institute for Social Research, College of Literature, Science, and the Arts, University of Michigan, 2448 ISR-Thompson 426 Thompson St, Ann Arbor, MI 48109 USA; 7 School of Social Work, College of Business, Health, and Human Services, University of Arkansas at Little Rock, 2801 South University Ave, Little Rock, AR 72204 USA; 8 Department of Pharmacy Practice and Psychiatry, University of Arkansas for Medical Sciences, 4301 W. Markham St, #522-4, Little Rock, AR 72205-7199 USA

**Keywords:** Implementation science, Early care and education, Childcare, Early intervention, Obesity, Child nutrition sciences

## Abstract

**Background:**

Despite the potential for Early Care and Education (ECE) settings to promote healthy habits, a gap exists between current practices and evidence-based practices (EBPs) for obesity prevention in childhood.

**Methods:**

We will use an enhanced non-responder trial design to determine the effectiveness and incremental cost-effectiveness of an adaptive implementation strategy for Together, We Inspire Smart Eating (WISE), while examining moderators and mediators of the strategy effect. WISE is a curriculum that aims to increase children’s intake of carotenoid-rich fruits and vegetables through four evidence-based practices in the early care and education setting. In this trial, we will randomize sites that do not respond to low-intensity strategies to either (a) continue receiving low-intensity strategies or (b) receive high-intensity strategies. This design will determine the effect of an adaptive implementation strategy that adds high-intensity versus one that continues with low-intensity among non-responder sites. We will also apply explanatory, sequential mixed methods to provide a nuanced understanding of implementation mechanisms, contextual factors, and characteristics of sites that respond to differing intensities of implementation strategies. Finally, we will conduct a cost effectiveness analysis to estimate the incremental effect of augmenting implementation with high-intensity strategies compared to continuing low-intensity strategies on costs, fidelity, and child health outcomes.

**Discussion:**

We expect our study to contribute to an evidence base for structuring implementation support in real-world ECE contexts, ultimately providing a guide for applying the adaptive implementation strategy in ECE for WISE scale-up. Our work will also provide data to guide implementation decisions of other interventions in ECE. Finally, we will provide the first estimate of relative value for different implementation strategies in this setting.

**Trial registration:**

NCT05050539; 9/20/21.

Contributions to the literature

- This study will capture implementation outcomes, health outcomes, and implementation costs for Together, We Inspire Smart Eating in early care and education. Measuring this combination is rare but necessary to optimize feasible implementation approaches for real-world replication.
- The enhanced non-responder trial design of this study will allow documentation of what strategies work for which sites and on what timeline.
- Our mixed methods evaluation will examine contextual factors associated with response to the adaptive implementation strategies and test underlying mechanisms proposed by the iPAIRHS framework.

## Background

Excess weight is linked with higher risk of 13 cancers [1], and the US has the highest rate of cancer attributable to body mass index (BMI) [2]. Dietary habits and weight trajectories in early life predict later health outcomes [3, 4]; thus, obesity prevention efforts must target young children. Specifically, children are 5 times more likely to be overweight or obese in adulthood if they are overweight in preschool [5]. On average, 60% of US children under age 5 have at least 1 non-parental childcare arrangement per week [6]. This equates to about 15 million children. Children spend 36 h a week in ECE settings, on average [7]. Thus, the early care and education (ECE) environment may be a prime setting to contribute to obesity prevention. Despite the potential for ECE to promote healthy habits, a gap exists between current practices and evidence-based practices (EBPs) [8].

Consistent with World Cancer Research Fund (WCRF) recommendations [9], Together, We Inspire Smart Eating (WISE) aims to increase children’s intake of carotenoid-rich fruits and vegetables (FV). WISE was co-developed with end users to meet the curricular and budgetary needs of the ECE context [10, 11] and is included in the US Department of Agriculture SNAP-Ed toolkit [12]. Research supports each WISE EBP: (1) multiple hands-on exposures to FV support food acceptance [13–19]; (2) role modeling by educators allows children to observe a trusted adult eating FV [20–22]; (3) positive feeding practices support children’s self-regulation [22–24]; and (4) mascot use associates a familiar character with FV [25–30]. Each EBP aligns with the Academy of Nutrition and Dietetics’ “Benchmarks for Nutrition in Childcare.” [31] Evidence also supports WISE as a whole [32, 33]. Compared to usual education, WISE increased FV intake [32] (8% increase in healthy carotenoid levels; 4% decrease in unhealthy range) [33]. Also consistent with WCRF guidance [9], parents reported significantly decreased fast food and sugar-sweetened beverages intake after a year of WISE [32, 34]. Thus, WISE has a positive impact in areas related to child obesity and adult cancer risk.

Standard approaches to WISE implementation have resulted in challenges and suboptimal fidelity to EBPs [35]. Little research exists to guide solutions. For example, although studies have demonstrated that implementation strategies can promote policy implementation (e.g., menu offerings) and improve the environment (e.g., access to water) [36], few studies have assisted educators to implement EBPs in ECE [37]. Further, no available studies report on implementation mechanisms in ECE [37, 38] (how *and* why strategies work *for whom*) or on cost-effectiveness of implementation strategies in ECE. Thus, practitioners lack data to drive decisions on EBP implementation in ECE. A prior small-scale trial by the study team demonstrated that stakeholder-selected implementation strategies were successful at improving fidelity to WISE EBPs, organizational readiness for change, and perceived appropriateness of the intervention [39]. This strategy cost $261 per classroom beyond intervention costs. These data suggest the package of stakeholder-selected strategies was effective for improving WISE EBP implementation. Yet, we do not know if all sites require all strategies to succeed. Scaling all strategies to all sites may be too resource- and time-intensive for wide dissemination [40].

The integrated Promoting Action on Research Implementation in Health Services (i-PARIHS) framework posits that components of successful implementation include characteristics of the innovation (the EBPs), recipients, context, and facilitation (i.e., implementation support) [41]. Successful implementation takes place when facilitation promotes the acceptance and use of an innovation based on the recipients’ and context’s needs. Facilitation exists along a continuum [42]. On one end, task-focused support provides technical and practical help. On the other end, holistic facilitation provides enabling support to cultivate shared meaning, connected networks, and personal development [42]. A central tenant of i-PARIHS is that successful implementation requires different levels and kinds of facilitation depending on characteristics of the innovation, the context, and recipients. The i-PARIHS framework guides our proposal in several ways. Our prior work identified determinants of WISE EBP implementation by applying i-PARIHS. These determinants guided engagement with stakeholders to select and tailor the proposed implementation strategies. Stakeholders prioritized *facilitation as a key strategy* to improve WISE EBP implementation, and we will tailor facilitation to reflect recipient and contextual needs. While i-PARIHS is ideal to inform implementation strategy tailoring, research has not tested it in this way. Further, i-PARIHS has received limited tests of underlying mechanisms [43–45], with most studies in health care [46, 47].

Consistent with i-PARIHS, adaptive implementation strategies reflect that a one-size-fits-all approach may not serve all settings well [48]. Not all sites may need all strategies; giving sites more than they need is expensive and wasteful. An adaptive implementation strategy provides decision points and tailoring variables to optimize resources. Table 1 presents the design features of an adaptive implementation strategy. In sum, an adaptive implementation strategy provides a “replicable guide” for *who* gets *what* implementation support and *when* [49]. Such a guide, if effective for optimizing implementation, would provide practical information for serving settings like ECE with limited resources.

**Table 1:** Adaptive implementation strategies design features

Design Features	Definition
Crucial decision points	Which strategies to begin the study (i.e., low-intensity)
	How and when response is measured
	What strategies are given to non-responders (i.e., high-intensity)
Tailoring variables	Measurement to identify non-responders and inform strategy intensity

The overall objectives of this study are to determine the effectiveness and cost-effectiveness of an adaptive implementation approach to improve adoption of the EBPs of WISE while also examining implementation mechanisms. Using a mixed methods enhanced non-responder trial, we will execute the following aims:

### Specific aim 1. Determine the effectiveness of an adaptive implementation strategy that tailors the intensity of implementation support versus a low-intensity strategy

We will compare the effect of continuing low-intensity strategies vs. augmenting with high-intensity strategies. We hypothesize that sites receiving high-intensity strategies will outperform sites continuing the low-intensity strategies on the primary outcome of intervention fidelity and on secondary child health outcomes.

### Specific aim 2. Examine moderators and mediators of implementation outcomes in a mixed-methods design

We will test organizational readiness and teacher experience as moderators of response to the implementation strategies. We will test educators’ perceptions of barriers, local implementation climate, and implementation leadership as mediators of the effect of the strategies on implementation outcomes.

### Specific aim 3. Assess the incremental cost-effectiveness of the adaptive implementation strategy

We will estimate the cost per unit of fidelity associated with the adaptive implementation strategy versus continuing low-intensity strategies. Results will also determine the incremental cost-effectiveness of applying the adaptive implementation strategy for improving BMI and other child health outcomes.

## Methods

### Study design

**Fig. 1:**
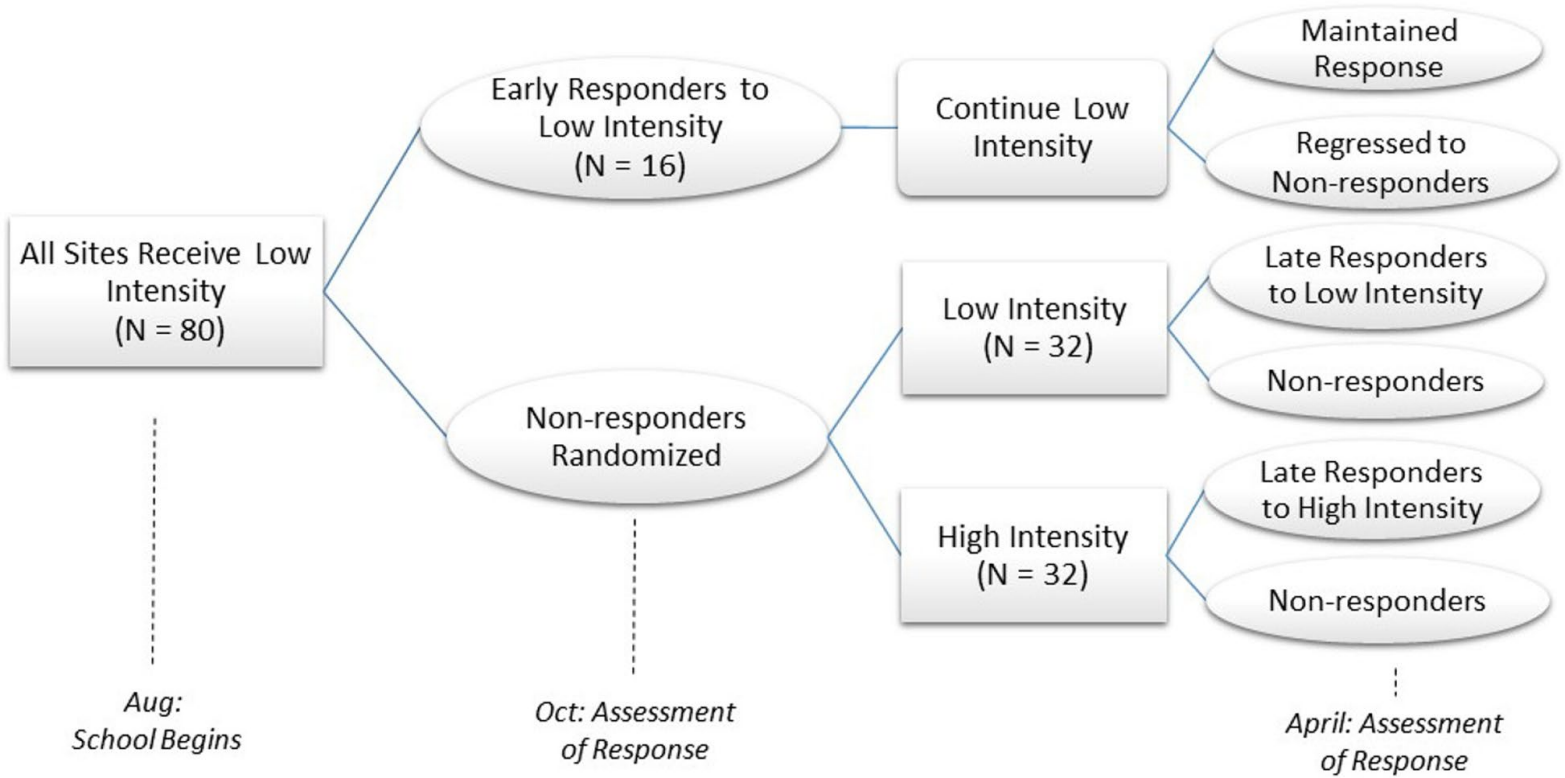
Cluster-randomized enhanced non-responder trial

We will use an enhanced non-responder trial [48] design to determine the effectiveness (Aim 1) and incremental cost-effectiveness (Aim 3) of an adaptive implementation strategy for WISE, while examining moderators and mediators of the strategy effect (Aim 2). In this trial, we will randomize sites that do not respond to low-intensity strategies to either (a) continue receiving low-intensity strategies or (b) receive high-intensity strategies (See Fig. 1). We will also use an explanatory, sequential mixed methods design (QUANT→qual) to provide a nuanced understanding of implementation mechanisms and contextual factors (Aim 2) [50, 51].

Decisions about key elements of our adaptive design were made based on stakeholder input. That is, educators who had received the intensive strategy package shared input through semi-structured interviews (*N* = 10). These interviews helped to delineate the low and high-intensity packages, define the tailoring variable, define non-response, and adjust timing of delivery of strategies and assessment of need for tailoring. The resulting adaptive implementation strategy package is specified as recommended by Proctor et al. [52] (Table 2). Low-intensity strategies include those that all sites receive at the beginning. High-intensity strategies are added at non-responders sites. Specifically, the facilitator receives data from the fidelity observation to coach and guide the educators in behaviors needed to support non-responders in achieving fidelity to EBPs.

**Table 2:** Specification of adaptive implementation strategy implementation approach

		Strategy	Actor(s)	Action	Temporality	Dose	Justification
Non-responders get addition of **HIGH INTENSITY**	Begin with **LOW-INTENSITY**	Obtain formal commitments	WISE facilitators & directors	Sign commitment after facilitators & directors discuss	Before educator training	One-time meeting	Address leadership buy-in
		Identify & prepare local champions [53, 54]	Appointed champion at each site	Provide 2-h training on navigating WISE	Within 2 months of training	1 training: contacts monthly & on request	Address capacity for change & climate supportiveness
		Develop implementation blueprint [55]	WISE staff with directors & champions	Provide target milestones & testimonials	With director before training	As desired	Give leaders tools to support change integration
		Remind educators [56, 57]	Classroom cutting board	Provide visual reminder of EBPs	At WISE lessons	As desired	Provide timely reminders
		Task- focused implementation facilitation [46, 47, 58, 59]	WISE facilitators, directors & champions	Provide support to directors & champions	Beginning 2 weeks after training	Monthly	Provide practical & technical help to site leader & champion; task & goal focus [42]
		Holistic individualized facilitation [46, 47, 58, 59]	WISE facilitators & educators	Provide in-person visits to directors, champions & educators	Beginning 2 weeks after training	Twice monthly; more upon request	Create and connect networks; holistic/ enabling focus on educators [42, 60]
		Distribute tailored educational materials [61–65]	WISE facilitators, champions, & educators	Provide educators with tailored EBP education	Each quarter	Varies by educator (up to 8)	Address barriers of beliefs, knowledge and skills for EBPs

Tailoring variable = classroom WISE fidelity observation in October

Non-responders = sites with fewer than 60% of classrooms meeting fidelity for 3 of the 4 WISE EBPs

Community partnership is key to reduce cancer-related health disparities [66, 67]. To this end, we will draw on Evidence-Based Quality Improvement (EBQI) methods throughout our study [68–70]. This process will develop researcher–stakeholder partnerships for joint decision making [68–74], consistent with Community-Engaged Dissemination and Implementation principles [75]. Our EBQI panel will include educators, directors, and staff from ECE as well as state policy leaders who can inform WISE scale-up. For example, at least 3 professional networks could use the adaptive approach we develop: Child and Adult Care Food Programs (CACFP) sponsors, Childcare Resource and Referral (CCR&R) agents, and USDA (United States Department of Agriculture) Cooperative Extension agents. The panel will meet 3 times per year and provide input into study recruitment, roll out of study protocol, interpretation of findings, and future planning. We will also disseminate our results to participants and stakeholders through infographic-style summaries and presentations at community events.

### Setting

#### Site selection

Sites will be from 4 geographic regions: Central AR, AR River Valley, North Central LA, and Southeast LA. A site is one ECE location; a site may have multiple classrooms with up to 20 children per classroom. A director provides leadership at each site; educators implement WISE in their classrooms. Sites within a 100-mile radius of staff offices; participating in CACFP and the state’s quality rating system; serving 15+ children ages 3 to 5 years; agreeing to participate in data collection; and not currently using WISE can be included. We will exclude sites unwilling to adopt WISE for all classrooms. Focusing on CACFP will maximize equitable reach, generalizability, and study impact. Most importantly, CACFP is a federal system that serves 3 million children per year [76], “targeting benefits to those children most in need.” [77]

We will recruit 80 sites, 40 in Arkansas (AR) and 40 in Louisiana (LA). We expect recruited sites will be diverse in Quality Rating and Improvement Systems scores [78], and we will model ratings in our analyses. We will recruit sites in 3 cohorts, 25–28 sites per year in 3 school years (across Y1-Y4). We will pool data across years for analyses and we will include cohort assignment as a control variable. A cohort-based design will allow us to limit the number of sites to 4 or less per facilitator per year—a number for which prior work indicates the greatest effect [46].

#### Classroom and child inclusion

All classrooms at a site will receive the same implementation strategies and participate in data collection. This reflects stakeholder input that sites would not participate unless all classrooms are treated equally. For primary analyses, we will include only classrooms that are non-responders (i.e. responding classrooms at non-responding sites will be excluded). In this way, we avoid contaminating analyses with classrooms that respond to the low-intensity strategy against the site trend. Based on our ECE experience, we expect 3 non-responding classrooms per site, on average; we have powered accordingly. Thus, we plan to include 192 total classrooms at 64 sites in our primary analyses. We will select one classroom at random per site to participate in collection of child outcomes (*N* = 64 classrooms, 15 children per classroom = 960 children total). Parents will provide consent.

#### Site randomization

The study will be cluster-randomized at the site level; we will randomize non-responder sites (i.e., those where < 60% of classrooms are not meeting fidelity standards). In our prior small-scale implementation trial, about 10% of classrooms were responders by the fall assessment (achieved fidelity to 3 of 4 EBPs). Considering these factors and that this study is larger and more diverse than our prior work, we anticipate at least 20% of sites will be responders after 2 months, leaving 80% of sites as non-responders for randomization. Higher non-response rates would improve statistical power. Sites will be randomized in a 1:1 ratio to the low-intensity or high-intensity strategies consistent with procedures of minimization and balancing [79, 80]. First, the balance between groups on potential confounding factors (e.g., site size, number of non-responder classrooms, demographics) will be examined. Then, we will randomly select one balanced assignment from the list of random assignments, which will balance sites on key factors while preserving advantages of randomly assigning sites [79, 80].

### Aim 1

#### Aim 1 measures

Table 3 presents the Aim 1 data collection plan. Measures align with Proctor’s Outcomes for Implementation Research taxonomy [81]. The school year calendar informs measurement timing. The primary outcome is fidelity to the WISE EBPs at the classroom level, using the WISE fidelity observational measure [82]. The measure includes 2 to 3 items per EBP on a 1 to 4 scale to receive an average, continuous fidelity score with 4 representing the highest fidelity. For each item, values are anchored to concrete, observable behaviors. Trained and field-reliable staff blinded to the study condition will collect fidelity data consistent with published protocols [77]. Secondary implementation outcomes are adoption as well as acceptability, feasibility, and appropriateness of WISE and the implementation strategies [61]. We will collect secondary outcomes through self-report from educators on the schedule in Table 3. The WISE delivery survey [35] captures the number/content of lessons delivered and material dissemination to parents. In the next school year, we will assess EBP sustainment (i.e., delivery and fidelity 12–18 months after initial implementation).

**Table 3:** Measures and data collection plan

Constructs	Measures	Frequency
Fidelity	WISE fidelity [82]	Oct, Jan, Apr of school year
Acceptability, feasibility, appropriateness of innovation *and* strategies	Weiner et al. pragmatic measures [61]	Aug, Jan, Apr of school year
Adoption	WISE delivery survey [35]	Monthly during implementation year
Sustainability	WISE delivery [35], WISE fidelity [82]	Fall of following school year
Child health outcomes	RRS [83], BMI [65], consumption [84]	Aug & Apr of school year

To measure the effect on child health outcomes, we will use Resonance Raman Spectroscopy (RRS), which measures skin carotenoid levels as a biomarker for colorful FV intake [62] with an optical hand scan [63, 83]. RRS reflects intake over the prior 4 weeks and is sensitive to individual differences and experimental changes [64, 85]. Trained staff will assess BMI with a standardized protocol [65] and interpret the data with 2000 CDC growth charts [86]. Finally, we will observe children’s target food intake with a standardized protocol used by our team in prior studies [84]. We will weigh food portions (to the nearest 0.1 g) before and after observation.

##### Implementation processes

First, site leadership will meet with WISE facilitators to discuss the formal commitment and implementation blueprint. Next, all staff will receive WISE training. At training, educators will receive the “reminder cutting board,” showing the 4 WISE EBPs for use during lessons. Next, sites will select a “champion” to be a liaison between the site and WISE facilitator. Champions receive standardized training to navigate WISE implementation before September.

In the low-intensity group, WISE facilitators will provide monthly task-focused facilitation targeted to site directors and champions. Facilitators in the low-intensity group will monitor implementation, identify and solve problems related to contextual barriers, and assist with navigating structural changes needed for WISE. In the high-intensity group, WISE facilitators will provide enabling, holistic facilitation tailored to the needs of the educators twice per month and more upon request. Facilitation in the high-intensity group will support educators in a one-on-one fashion, helping to set goals, fostering peer networking, developing shared vision among leaders and staff, and building meaningful relationships that support change efficacy. This will include the provision of tailored educational materials and coaching based on observed fidelity reports.

Each study region will have 2 trained facilitators with experience in the ECE setting and/or WISE. Further, all facilitators will receive standard training and toolkits (e.g., sample scripts, testimonials, motivational interview examples). This is based on the Veterans Health Administration Implementation Facilitation Training [87], which 2 study staff completed in 2019. This training has been adapted for WISE and condensed for delivery in a 4-h session. After training, new facilitators will accompany experienced facilitators for 2 field visits to observe. The new facilitators will lead at least 2 visits with support and feedback from the experienced facilitator. Facilitators will take part in monthly reflective supervision calls led by the PI aimed to process experiences in the field, sharing lessons learned, and collaborating on ideas for supporting sites and teachers. All facilitators will log their activities (e.g., visits, calls, emails, champion contacts). The PI will compare the facilitator logs against the core implementation facilitation activities checklist for fidelity monitoring [88] and provide corrective guidance as needed.

#### Aim 1 analyses

We will manage data with REDCap [89], a secure, web-based electronic data capture tool hosted at UAMS. For Aim 1 analyses, analysts will examine data for missing values, extreme scores, and variable distributions. We expect missing values on the primary outcome to be minimal because study staff will collect these data. If the missing values percentage exceeds 5% [90], we will use an appropriate method for using all available data such as multiple imputation or full information maximum likelihood (FIML) estimation for analyses. For our primary analysis, we will use linear mixed-effects regression models [91] to test for group differences in fidelity outcomes at the school year end, while accounting for classroom nesting within site. Covariates will include state, site size, cohort, turnover rate, October fidelity, quality rating, and demographics. The statistical significance of the treatment group predictor (α = .05) will be used to determine significant differences in fidelity outcomes for the low- vs high-intensity groups. Additional analyses will include repeated outcomes from all time points to test for treatment group differences across time and time-by-treatment effects. We will repeat these analyses for secondary implementation outcomes. We will also examine child-level outcomes using linear mixed-effects regression models, which account for a child’s nesting within classrooms and sites. Parallel to primary analyses, we will first test treatment group differences at the spring assessment and then examine treatment and time-by-treatment effects using all time points. For all analyses, a significant, positive effect of treatment group will support the effectiveness of applying high-intensity strategies at sites that do not respond to low-intensity strategies initially. An exploratory analysis will describe the number of sites that were early responders and maintained response until the April assessment (versus regressing to non-responders over time).

For power analysis, we used Optimal Design software [92] to accommodate the clustered design of classrooms nested in sites. Our estimated sample size is based on the primary fidelity outcome and is analogous to powering a 2-arm randomized controlled trial. We have powered our study to detect a practically meaningful 1-point difference on our fidelity scale: 1 point would differentiate an educator who implements a practice only somewhat (e.g., score of 2) from an educator who implements a practice to a significant degree (e.g., score of 3). Based on standard deviations from prior small-scale trial, a 1-point difference would yield Cohen’s d effect sizes between .83 (Mascot) and 1.68 (Role Model). Assuming 64 non-responder sites (assigned 1:1) with an average of 3 non-responding classrooms per site (192 classrooms), the largest previously observed 0.20 Interclass Correlation Coefficient (ICC), and 2-sided α == .05, we will have 80% power to detect an effect size of d = 0.49 or larger. We do not anticipate site-level attrition, but even with ~ 20% attrition (*N* = 50 sites), we would have 80% power to detect an effect size of d == .56 or greater. Assuming one randomly chosen classroom per site, 15 children per classroom (*N* = 64*15 = 960), a 0.10 ICC (largest observed child-level ICC in the prior small-scale trial), and 2-sided α == .05, we will have 80% power to detect an effect size of d == .29 or larger for child-level outcomes, which corresponds to an effect size of between small and medium [93].

### Aim 2

#### Aim 2 quantitative measures

**Fig. 2:**
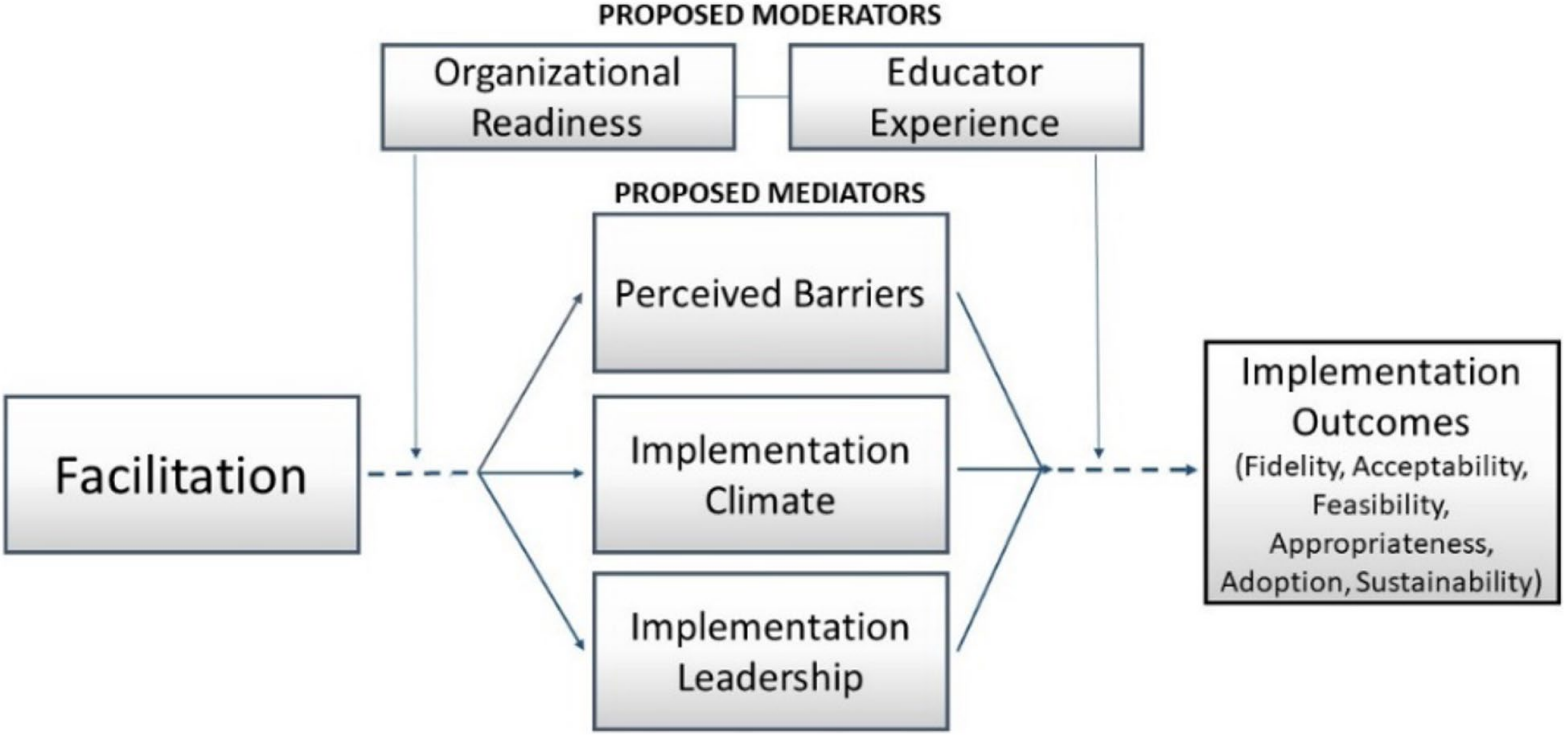
Conceptual model of proposed moderators & mediators

Quantitative analyses will test 2 moderators and 3 mediators specified a priori (See Fig. 2). During the baseline period (prior to October), educators at participating sites will complete assessments of potential moderators and mediators. Educators will also complete surveys mid-year and at the school year end to assess proposed mediators. We expect 2 educators per classroom to complete the survey, 6 per site on average. This follows the best practice of assessing moderators before randomization [94, 95] and assessing mediators at a minimum of three points in time [96, 97]. Further, our design includes key features to establish causal inference including temporality and experimental manipulation of dosages of facilitation [98]. Educator responses will reflect site experiences, and we will aggregate educator responses to the site level for analyses. The research team will collect these data in person with paper surveys or emailed survey links (reflecting technology access and use in ECE). We will capture data with REDCap for secure storage. All classroom staff (lead and assistants) will complete assessments.

##### Moderation

Moderation measures will include The Organizational Readiness to Change Assessment (ORCA) [99], which we adapted and tested in ECE in our prior work. For this study, we will focus on the context subscale, which is consistent with our focal moderator and based on i-PARIHS. The baseline survey will also capture educator background including years of experience and type/ frequency of prior nutrition and feeding training to create a composite educator experience variable.

##### Mediation

First, we hypothesize that facilitation will decrease the perceived barriers to implementation (e.g., by helping to identify problems and solutions) [42]. Second, we expect that facilitation will improve implementation climate (e.g., by developing shared meaning [42], assisting with boundary navigation, and supporting role clarity [42, 100]). Finally, we hypothesize that facilitation will improve implementation leadership and the knowledge and behaviors leaders leverage to support EBP implementation [101] (e.g., by navigating group interests, modeling empowerment, and building organizational structures [42, 100]). Based on the recommended process by Lewis et al. [102], Fig. 2 presents our proposed model that links facilitation to the proposed mediators (proximal outcomes) and targeted implementation outcomes (distal outcomes). Facilitation (e.g., dose, target) will be measured using the facilitation logs described in Aim 1; however, we will conceptualize facilitation dichotomously for analyses (high and low-intensity). We will apply widely used and validated measures of proposed mechanisms, including the Implementation Climate Scale [103] and Implementation Leadership Scale [101], recently adapted for educational settings [104]; the perceived barriers measure [105] is a checklist of challenges educators reported in our formative work was used in our prior work.

#### Aim 2 quantitative data analyses

Analyses will include 64 sites randomized (1:1) to the low- or high-intensity strategies. Moderator analyses will be conducted using mixed effects logistics regression models with a treatment main effect (low- vs high-intensity), the moderator main effect (organizational readiness and educator experience), and the interaction between the two. The interaction term significance (α = .05) will be evaluated to test moderation. Models will account for the classroom nesting within sites and include controls for state, quality rating, and key demographics. For mediation analyses, we will test a multilevel, multiple mediator model in a Structural Equation Modeling (SEM) framework [106] to account for students clustering in classrooms and classrooms within sites. That is, all 3 mediators will be tested simultaneously. Specifying a multiple mediator model is less biased than testing single mediators one at a time [106–108]. Using 95% bootstrap confidence intervals [53], significance tests in SEM are also less biased than sequential hypothesis testing approaches to mediation tests [109]. Using data at three time points, we will be able to model that Y (independent variable) precedes M (mediator) in time, and M precedes Y (dependent variable) in time; prior levels of M and Y can be controlled.

#### Statistical power for secondary moderation and mediation analyses

For moderator analyses, we will have 80% power detect a Cohen’s *f* ^2^ of .13 which falls between a small (*f* ^2^ = .02) and medium (*f* ^2^ = .15) effect size [93]. In mediation analyses, the indirect effect is the product of 2 regression coefficients and is not distributed normally, which poses a challenge to power calculations [110]. However, Fritz and MacKinnon [111] recommend a bias-corrected bootstrap method for the indirect effect that, in our sample size of 64 sites, would provide 80% power to detect an indirect effect composed of 2 large-sized constituent effects, or a mix of a large-sized and a medium-sized effects.

#### Aim 2 qualitative measures

Qualitative methods will provide detail and elaborate on potential additional candidate moderators and mediators at a subset of purposively selected sties based on response type. We will use qualitative data to provide detailed understanding of response to low- and high-intensity strategies. Specifically, quantitative data from the enhanced non-responder trial will identify 5 categories of response to implementation strategies (Fig. 1): (1) early responders to low-intensity (by October), (2) late responders to low-intensity (by school year end), (3) non-responders to low-intensity, (4) responders to high-intensity, and 5) non-responders to high-intensity. Through purposive site visits, we will collect qualitative data within each response type. We expect to target 3 sites per response type to reach a total sample of 15 sites (split across state and study years). We expect to reach saturation with 15 sites, but we are prepared to increase to 20 if needed to reach saturation. During site visits, the research team will conduct semi-structured, key informant interviews with directors and focus groups with educators (4 to 6 educators per Krueger [50, 112]). This format is cost-effective and will allow educators to share experiences (independent of directors) [50]. Director interviews and educator focus groups will elicit perceived reasons why the strategies worked (or failed) at their site, practical strategies of leadership support, and relevant factors in the implementation climate. Concepts from the i-PARIHS framework will inform interviews and focus groups guides (Table 4). Additionally, the research team will capture field notes of the site activities, processes, and interactions that may influence response to the strategies.

**Table 4:** Sample interview questions by i-PARIHS constructs

Context	*What is it like to work at this center? How did that influence implementing WISE? How did your leadership get involved?*
Innovation	*Tell me about how WISE worked in your classroom.*
Recipients	*As you implemented WISE, what was most helpful to you? Least helpful?*
Facilitation	*Who was your WISE coach? How did you interact with them? What did the WISE coach do that helped? What do you wish they had done to better support you?*

#### Aim 2 qualitative data analyses

Qualitative analyses will focus on identifying similarities and differences between site response types. Transcripts will be matched with observed field notes and coded using directed content analysis [113]. The i-PARIHS framework will provide a template of sensitizing concepts to label significant, recurrent ideas [114], particularly ideas that suggest emergent candidate mediators and moderators. We will incorporate inductive codes as we identify additional salient factors [115]. Primary and secondary coders (at least 1 each from AR and LA) will code the same transcripts until inter-rater reliability is established. Minimum reliability will be set at Kappa of 0.75, which reflects excellent agreement between coders [116]. Coding will be independent after establishing reliability. Coders will hold weekly meetings to discuss iterative expansions to the codebook, to reach consensus about unclear codes and to document tentative patterns in the data. A third-party team member will resolve disagreements [117]. Participants and stakeholders will review site-level summaries of findings. We will conduct analyses of qualitative interviews yearly and use findings to revise the interview guide for subsequent interviews (e.g., identify probing needs, generate new questions).

##### Integrating findings

As we interpret Aim 2 findings, we will connect quantitative and qualitative data. This will include: 1) *expansion* of quantitative findings to provide detail through qualitative data and 2) *complementarity* to deepen understanding and identify other potential moderators and mediators not focused on in quantitative analyses. Thus, qualitative data will explain and elaborate on quantitative findings.

### Aim 3

#### Aim 3 measures

The cost-effectiveness analysis (CEA) will construct incremental cost-effectiveness ratios (ICER) to estimate the marginal differences in costs and in fidelity and child outcomes between the adaptive implementation strategy and continuing the low-intensity implementation strategy. We will calculate implementation strategy costs based on time and travel data collected in the facilitation log and known material purchase costs. Based on work by Ritchie et al. [118], facilitators will log all activities and travel time using REDCap, which was tested and found feasible in our prior work.

#### Aim 3 analyses

We will calculate the incremental cost-effectiveness ratio in 4 steps. First, we will use data from Aim 1 as estimates of fidelity and child outcome changes (i.e., BMI, RRS, target food consumption) for both study conditions. We will aggregate these findings to the site. Next, we will calculate the costs associated with implementation at each site. The WISE intervention cost is the same at all sites, and the ECE system does not accrue downstream costs or benefits. Therefore, we focus on implementation costs only, which comprise 4 categories (Table 5). These will be collected using a micro-costing approach, and expenses will be applied to the appropriate site.

**Table 5:** Implementation cost, source, and estimation methods

Cost category	Data source
Facilitator salary & benefits	Facilitation time tracking log; facilitator salary data from employer records
Facilitator travel expenses	Facilitator travel expense reports
Educational resources	Delivery logs & printing receipts
Other classroom & site resources	Facilitator resource delivery tracking

Then, we will estimate covariates to adjust for site-level differences in fidelity. We will use the same covariates used to control for site variation in Aim 1 and aggregate child-level covariates to the site. Incremental costs will be calculated using intent-to-treat analysis to estimate the effect of treatment allocation. We will use generalized linear models (GLMs) to estimate the effect of implementation intensity on fidelity, child outcomes, and implementation costs. We will compute 2 outcome predictions for each site based on the coefficients from the GLM regressions and the covariates for each analysis [119]. The first prediction will be as if the site was randomized to the adaptive strategy, and the second prediction will be as if the site was randomized to the low-intensity strategy. The difference between these predictions represents the incremental effect of the implementation strategy on fidelity, child outcomes, or costs. Lastly, we will calculate the incremental cost-effectiveness of adding the adaptive strategy relative to continuing the low-intensity strategy. The numerator will be the incremental difference in total implementation costs incurred at sites receiving the adaptive strategy compared to sites continuing the low-intensity strategy. The denominator will be the difference in the changes in fidelity or child outcomes between the fall implementation and spring implementation assessments for the adaptive strategy compared to sites continuing the low-intensity. We will use a nonparametric bootstrap with replacement method with 1000 replications to generate an empirical joint distribution of incremental implementation costs and fidelity or child outcome change scores*.* Analysts will build preliminary models using data from the first cohort (Y2) to promote analysis expedience when all three cohorts are completed (Y4).

## Discussion

Arkansas (AR) and Louisiana (LA) are among the states in the US with the highest obesity rates, lowest quality diets, and highest cancer rates [120]. Given the limited economic resources of these states, community systems need obesity prevention efforts that optimize resources through innovative, tailored implementation in settings serving community populations. ECE is a key real-world context for nutrition promotion and obesity intervention [31, 121, 122], but implementation gaps persist in this setting. Thus, this research has substantial potential to inform pragmatic, scalable guidance on the strategy intensity needed to implement and scale health-related EBPs in ECE.

This study has the potential to advance the field of implementation science in several ways. First, this study can contribute to a shift from one-size-fits-all implementation strategies to strategically sequenced implementation strategy packages. This is critical given that ECE, particularly programs in our study, serves children of minority and lower-SES status, populations at higher child obesity risk [123] and often have limited resources to serve children families. Our approach may help to optimize the minimum resources needed to achieve desired outcomes. This study also represents a significant contribution to testing theory and examining mechanisms in implementation science. Specifically, our examination of i-PARIHS suggested moderators and mediators will test the “encapsulated theory” implied by this framework [41]. This aspect of our work represents an emphasis on identifying the who, what, and how of facilitation as an implementation strategy and adds to the limited body of research using mixed methods to explore implementation mechanisms [ 38]. Finally, our research models implementation outcomes, health outcomes, and implementation costs for WISE in ECE. Measuring this combination is rare but necessary to optimize feasible implementation approaches [124].

At the conclusion of this study, we expect several tangible outcomes. First, we expect data from this study to serve practitioners in ECE (e.g., CACFP, CCR&R, and Extension), local agency directors, and state-level policy makers in allocating resources to implement EBPs and providing data on characteristics of sites that are likely to need higher-intensity support. Specifically, this study will provide crucial data to inform WISE scale-up and dissemination with applicable lessons for other interventions and contexts. Follow-up assessments will reveal how the implementation strategies affect WISE EBP sustainability in school years following the initial WISE launch. For example, we will be able to compare sustainability between sites that respond early to low-intensity strategies (by October assessment) and sites that respond late to low-intensity strategies (by school year end). Qualitative data from Aim 2 and quantitative data on fidelity in the sustainability assessment from Aim 1 will be linked to understand the specific ways low- and high-intensity strategies influence sustainability as well as characteristics of sites that achieve sustainment. According to a recent review, [125] fewer than 25% of funded R01s in Dissemination and Implementation Science studies have evaluated “the impact of a strategy on sustainability.”

This study will also provide unique knowledge for the implementation science field about facilitation. Our design will allow comparisons between the effect of a strategy bundle with narrowly focused facilitation and a more holistic, individualized facilitation. Further, we will be able to provide insight into mechanisms by which facilitation influences implementation [100, 126]. Our random assignment to different facilitation levels embedded in each strategy package, our use of multiple measurements of mediators at key time points, and our multilevel SEM approach will improve causal inference about the relationship between facilitation, the proposed mediators, and the targeted implementation outcomes [109, 127]. Thus, we expect to provide an important test of the i-PARIHS theory, while illustrating best-practices in mediation analyses in an implementation science study.

Finally, this study will provide important data on incremental costs for fidelity and child health outcomes. We will also explore costs of increasing implementation outcomes of potential interest to future implementers (e.g., acceptability, feasibility). This will inform future scale-up of our adaptive implementation strategy for relevant ECE stakeholders (e.g., education state departments, state and federal Head Start programs). Further, data on the cost-effectiveness implementation strategies in ECE programs receiving federal CACFP support (e.g., Head Start) can support policy for these government-funded programs. No available studies in ECE report on implementation strategy costs [37].

This study has potential challenges and limitations. A primary challenge is that staff turnover can be significant in ECE. At the low-intensity sites, facilitators would encourage local site champions to demonstrate a WISE lesson and follow up by providing resources and inviting questions; at the high-intensity sites, WISE facilitators would visit new educators’ classrooms during a WISE lesson to answer questions and identify resource needs. We will include turnover as a covariate in analyses. In addition, we may not have selected the most salient moderators and mediators for testing. However, parsimony and power considerations require us to specify theorized moderators and mediators a priori. For moderation, we have focused on two potential moderators with support in the literature and in our prior work. For mediation, we have focused on targets of holistic facilitation to determine if facilitation in the high-intensity strategy activates shifts in the climate and organization as proposed [42, 100]. We are not powered for examination of amplification effects (e.g., moderated mediation); nor is it a scientific focus of our study. Qualitative data will explain quantitative findings and elaborate on other potential moderators and mediators. If we do not find significant mediation effects, we can still assess if our strategy failed to produce the desired effects on the proposed mechanisms and/or if the proposed mechanisms failed to produce the desired effects on the targeted implementation outcome [127]. This distinction would inform future work, including strategy selection and theory refinement. Finally, prior studies are unclear on the relationship between early childhood BMI and quality of life, limiting the current study to an estimate of cost per unit of BMI change. While a generalizable measure of effectiveness (e.g., quality adjusted life year), may be preferable for comparison across interventions, it is beyond the scope of this study.

## Conclusions

We expect our study to provide an evidence base for structuring implementation support in real-world ECE contexts, ultimately providing a guide for applying the adaptive implementation strategy in ECE for WISE scale-up. Our work will also provide data to guide implementation decisions of other interventions in ECE through improving targeted application of strategy intensity to optimize resources. The results from this study will position us for future research to test the transfer of the adaptive implementation support away from the research team and to the ECE systems. This research path will advance us toward our long-term goal of EBP implementation in ECE to improve diet quality and health outcomes for children.

## Data Availability

The datasets used and/or analyzed during the current study will be available from the corresponding author on reasonable request.

## Abbreviations

ECE: Early care and education
WISE: Together, We Inspire Smart Eating
BMI: Body mass index
EBPs: Evidence-based practices
WCRF: World Cancer Research Fund
FV: Fruits and vegetables
i-PARIHS: Integrated Promoting Action on Research Implementation in Health Services
EBQI: Evidence-Based Quality Improvement
CACFP: Child and Adult Care Food Programs
CCR&R: Childcare Resource and Referral
RRS: Resonance Raman Spectroscopy
REDCap: Research and Evaluation Data Capture Application
ICC: Interclass Correlation Coefficient
ORCA: Organizational Readiness to Change Assessment
SEM: Structural Equation Modeling
GLMs: Generalized linear models
AR: Arkansas
LA: Louisiana
